# Analysing community-level spending behaviour contributing to high carbon emissions using stochastic block models

**DOI:** 10.1038/s41598-025-14364-7

**Published:** 2025-08-08

**Authors:** Ognyan Simeonov, Valerio Restocchi, Benjamin D. Goddard

**Affiliations:** 1 School of Informatics, University of Edinburgh, Informatics Forum, 10 Crichton St, Newington, Edinburgh, UK; 2 School of Mathematics and Maxwell Institute for Mathematical Sciences, University of Edinburgh, James Clerk Maxwell Building, Peter Guthrie Tait Rd, Edinburgh, UK

**Keywords:** Carbon emissions, Spending behaviour, Network science, Community detection, Green economy, Climate sciences, Environmental social sciences, Mathematics and computing

## Abstract

Large financial transaction datasets are increasingly used to estimate carbon emissions associated with individual spending. However, to effectively target high-emission spending areas and implement successful carbon reduction strategies, policymakers and financial institutions need to understand individual consumer spending behaviour. In this study, we describe an approach to identify spending patterns in large financial transaction datasets, using stochastic block modelling for community detection on a bipartite network. This is an effective method to form communities of consumers who share similar spending patterns across merchant categories, allowing us to identify the categories causing high carbon emissions for each group of consumers. We also introduce a modification to the weights of the bipartite network which allows us to keep the average community spending constant across different categories. The impact and applications of this study are twofold. First, it highlights the importance of transaction datasets and stochastic block modelling in providing insights for financial institutions in their efforts to decarbonise by identifying areas for targeted behavioural strategies for carbon reduction. Second, it provides researchers with a framework to examine how different factors, such as consumer spending patterns, energy usage, or transportation habits, interact with one another. This is done while keeping overall spending levels consistent across various communities, allowing for a controlled analysis of behavioural and economic impacts on carbon reduction efforts.

##  Introduction

Reducing carbon emissions has recently become one of the primary goals of policymakers and institutions around the globe. Countries in the Carbon Neutrality Coalition are currently in the process of building and implementing plans to reach net-zero emissions by 2050, with one of the United Nations Sustainable Development Goals highlighting the importance of a sustainable and resilient future^1^.

In an effort to track emissions associated with individual expenditure, financial and government institutions have developed carbon calculators to inform consumers about their daily emissions^2–5^. While the methodology behind most of the company-specific carbon calculators is proprietary information and not accessible to the general public, Trendl et al.^6^ recently published a widely accessible open approach for calculating carbon emissions from micro-level transaction data. The authors develop category-specific carbon multipliers based on data from the UK Living Cost and Food Expenditure (LCFS) survey and the UK Multi-Region Input-Output (UKMRIO) greenhouse gas emission values. They show that financial transactions present a reliable alternative to traditional survey-based methods for carbon emissions tracking and published statistics from their transaction dataset, making the study a good benchmark for comparison.

Identifying consumer groups with similar spending and associated carbon emissions patterns is crucial for designing effective interventions that encourage sustainable consumption. These insights can inform policies at both governmental and industry levels. Policymakers can use this information to develop targeted sustainability initiatives, such as tax incentives or subsidies for low-carbon purchases, while financial institutions and businesses can design personalised incentives to reduce carbon emissions based on consumer profiles^7,8^. However, there remains an open challenge in understanding how such consumption patterns can be used to inform the design of effective, group-level carbon reduction behavioural interventions.

Since financial institutions and governments work with datasets covering millions of consumers, tracking spending behaviour on an individual level and tailoring interventions to each consumer is highly impractical. Instead, the ability to identify large consumer groups with shared spending habits and emissions profiles is essential for the scalable implementation of behavioural incentives that promote sustainable spending. To achieve this, clustering algorithms can be used to systematically group consumers based on their transaction data, facilitating the design of targeted and scalable interventions that align with the shared characteristics of these groups.

### Related work and our contribution

Several studies have examined the role of clustering algorithms in uncovering groups of consumers with similar habits. Traditional clustering methods have been widely used to segment consumers based on their spending behaviours.

For instance, de Vries, Carlson & Moscato^9^ introduced a clustering approach based on a Minimum-Spanning-Tree and k-Nearest Neighbour (MST-kNN) graph, successfully segmenting online consumer behaviour into five distinct groups, each associated with unique engagement patterns. Sgroi et al.^10^ applied a cluster analysis approach to study consumer trends in functional foods, showing that demographic factors such as age, gender, and income play significant roles in shaping spending behaviour. Additionally, Chang, Hung & Ho^11^ proposed an anticipation model combining clustering analysis with association rule mining to predict potential customers’ purchasing behaviours based on their interactions with web platforms.

Financial transaction data has also been widely used to study consumer spending patterns. Di Clemente et al.^12^ investigated the sequential nature of consumer transactions, finding that purchase sequences of Merchant Category Codes (MCCs) follow a Zipf-like distribution and can be used to cluster consumers into groups with similar socio-economic characteristics. Dong et al.^13^ explored how work and home environments shape purchasing behaviours, showing that spending habits are influenced by social structures and geographic proximity. More recently, Bahrami et al.^14^ leveraged machine learning techniques to predict customer spending behaviour based on transaction histories, demonstrating the predictive power of financial transaction networks.

Clustering techniques have been employed to identify consumer groups with environmental considerations. For example, Jansson, Marell & Nordlund^15^ performed a cluster analysis on Swedish car owners and identified three distinct consumer segments based on their pro-environmental purchasing behaviours. However, most studies in this area focus on a singular type of purchasing behaviour—such as car ownership, energy consumption^16^, or organic food purchases^17^—without accounting for broader spending patterns. Moreover, these studies often rely on survey data or self-reported behaviours, which may not accurately reflect real-world spending. In contrast, we work with transaction datasets, providing a more comprehensive and objective view of consumer spending habits across multiple categories. This allows us to capture interactions between different types of purchases, offering a more holistic understanding of sustainable consumption.

Recent work by Wells et al.^18^ has shown the potential of using financial transaction data to segment households based on carbon footprints. This study uses k-means clustering on over 700,000 customers, incorporating socioeconomic indicators along with emissions by categories to identify household typologies for targeted retrofit interventions. While this study represents a significant step forward, the approach focuses on overall customer segmentation and relies on demographic attributes and distance-based clustering.

In contrast, our study focuses on a sustainability-oriented subset of the population, analysing their spending behaviour and proposing a complementary approach that clusters customers solely based on their transaction patterns across categories, without relying on socio-demographic data. Including such demographic or geographic variables as features during clustering can inadvertently reinforce pre-existing societal structures or inequalities, leading to clusters that may reflect known groupings. Instead, we apply a Stochastic Block Model (SBM) community detection method on a bipartite network of customers and merchant categories, where edges represent the proportion of spending and associated carbon emissions.

To the best of our knowledge, our work is among the first to apply SBM to financial transaction data in the context of sustainability. Unlike traditional clustering techniques, SBM identifies communities based on network structure rather than predefined attributes, mitigating potential biases related to demographics or geography. Moreover, SBM has been shown to be particularly effective at uncovering statistically significant latent structures in behavioural networks, as it models the generative process of link formation directly rather than relying on distance-based heuristics^19^. Along with its probabilistic foundation, high resolution limits, and ability to capture hierarchical cluster structures, SBM is particularly well-suited for identifying meaningful communities in behavioural networks.

This approach groups consumers with similar overall emission profiles, rather than focusing on a single category in isolation. By doing so, we can identify consumer segments that contribute to carbon emissions in comparable ways and develop targeted interventions to encourage sustainable spending habits. This network-based methodology provides a more comprehensive understanding of consumer behaviour, allowing for more effective policy design and personalised strategies to reduce emissions at scale.

The rest of this paper is organized as follows. In Section “Data and methods”, we explain the financial and government datasets used for this study. We also describe the bipartite network creation method and the SBM approach used to identify groups of customers with similar spending behaviour. In section “Results”, we present results of the community detection algorithm and analyze the effects of modifications to the edge weights and category groupings. Section “Discussion” provides a comparison of our results to the literature and discusses the importance and limitations of our study. Section “Conclusion” presents our conclusions.

## Data and methods

We obtain financial transaction data from ekko^20^, a sustainable banking FinTech company, alongside several government datasets. These include the Index of Multiple Deprivation (IMD)^21^ , Living Cost and Food Survey (LCFS)^22^, and UK Multi-Region Input-Output (UKMRIO) data^23^. These datasets provide socio-economic context and environmental impact metrics for the customers of the banking initiative. In this section, we describe the dataset and also outline the network construction approach and community detection methodology, aimed at uncovering patterns in consumer behaviour and carbon emissions associated with everyday spending. The code used to apply the methodology for this research is freely available ^24^.

### Financial transaction dataset

We obtained a debit card transaction dataset from ekko^20^, a sustainable banking FinTech we partnered with, containing tens of thousands of transactions spanning from 2021 to 2023 from 1,362 customers based in the UK. A distinctive feature of these customers is their higher level of environmental consciousness compared to the general population. This is demonstrated by their engagement with ekko, which focuses on promoting environmentally friendly practices and rewards customers for their transaction activity. These rewards are specifically designed to help customers consider the carbon footprint of their everyday transactions. The FinTech’s mobile application allows customers to view in real-time the environmental impact of their spending, along with personalized insights and streamlined features that make it easier to adopt greener choices in their daily lives.

The financial transaction dataset includes key customer metrics such as customer ID, postcode, age, and transaction details including transaction time, Merchant Category Code (MCC), and amount spent. We summarize the age and IMD distributions of the customers and discuss how they compare to national levels in Appendix 1.

The spending categories of customers in our analysis are defined by the Merchant Category Codes (MCCs) assigned by the debit card provider, in this case, Mastercard. Each transaction is linked to a merchant category, described by spending type. An extensive list of merchant categories and the transactions that fall within each is available in Mastercard’s reference documentation^25^.

However, it is important to note that some spending categories are incompletely represented in the dataset due to the nature of the available transaction data. In particular, utility payments are frequently made via direct debit, which is not included in this dataset. As a result, spending in the MCC 4900 category (“Utilities—Electric, Gas, Heating Oil, Sanitary, Water”) is less than expected. Other services commonly paid via direct debit, such as subscriptions (MCC 4899—“Cable, Satellite, and Other Pay Television and Radio Services”) and rent payments (MCC 6513—“Real Estate Agents and Managers—Rentals”), are also underrepresented in this data. This limitation should be kept in mind when interpreting the absence of energy-related or subscription-based spending and emissions in the figures and cluster analysis.

**Fig. 1:**
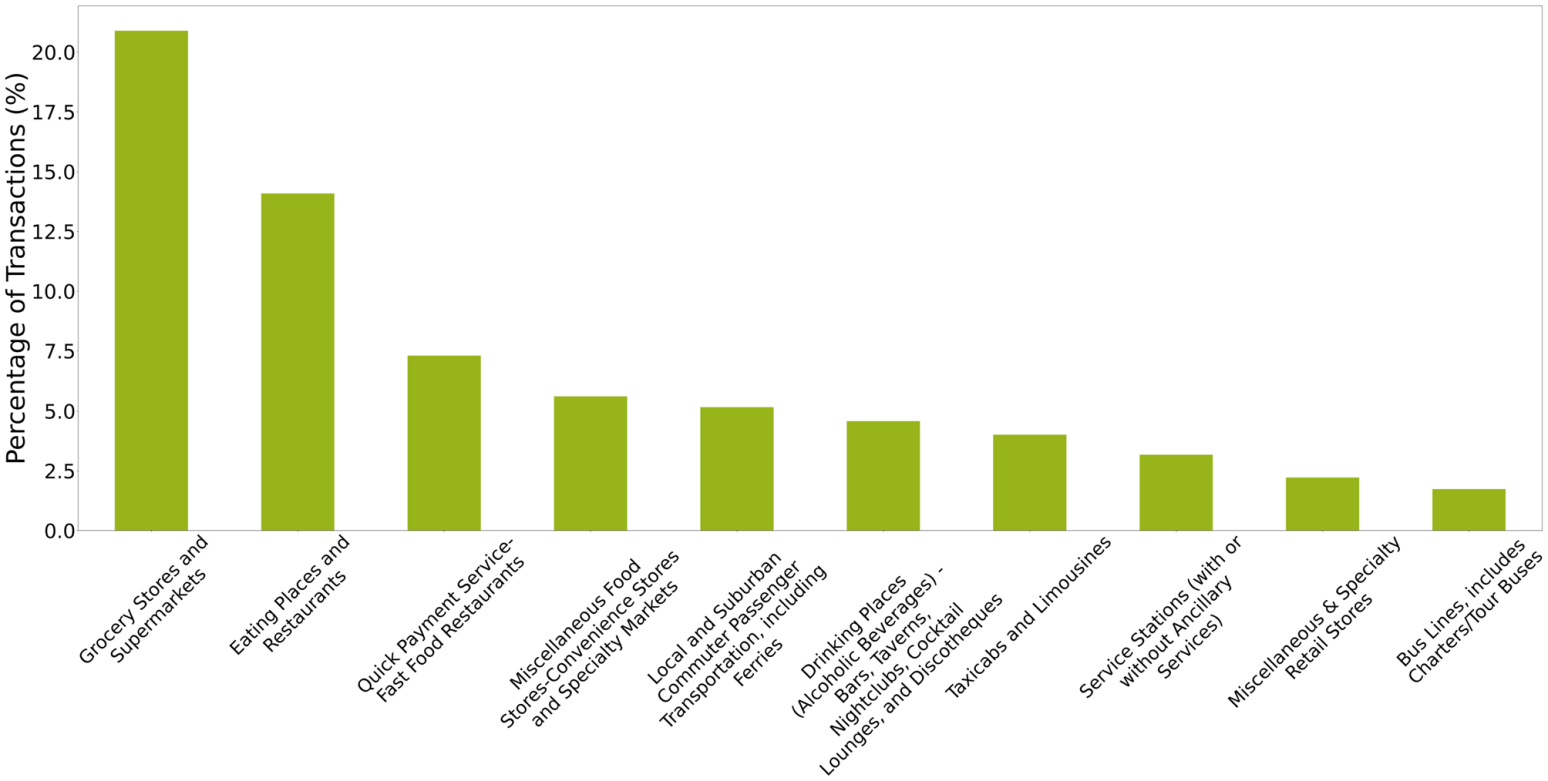
Percentage of transactions in the top 10 most frequent Merchant Category Codes (MCCs) across the entire sample (N = 272 customers).

For the Stochastic Block Modelling (SBM) analysis, we focus on customers who have made a minimum of 30 transactions across 10 distinct categories to ensure we capture consistent and diverse everyday spending patterns. This threshold is set to include only those customers with a sufficiently broad spending behaviour, preventing the inclusion of infrequent or niche spenders that could skew the analysis. With this criterion, we retain a sample of 272 customers, ensuring statistical significance. In Fig. 1 we present the percentage of spending behaviour across MCCs of this sample. To further validate our approach, we apply the model to larger, artificially generated transaction datasets in subsection *Applying the model on larger dataset*. The results show that although the set of customers may vary with different minimum thresholds for transactions and MCCs, the use of stochastic block modelling still yields clusters with consistent underlying patterns (see subsection *Applying the model on larger dataset* for more details). The choice of thresholds does not fundamentally alter the overall structure uncovered by the analysis, but it does change the specific composition of the clusters based on the number of customers we allow for.

### Government datasets: IMD, LCFS, and UKMRIO

This study relies on several openly available government datasets to contextualise spending patterns and their associated carbon emissions within broader socio-economic and environmental contexts. The datasets used are the Index of Multiple Deprivation (IMD), the Living Cost and Food Survey (LCFS), and the UK Multi-Region Input-Output (UKMRIO) data.

The Index of Multiple Deprivation (IMD) provides a detailed measure of deprivation at a small area level across England, covering indicators such as income, employment, education, health, crime, housing, and the living environment. We use the English indices of deprivation 2019 dataset, published by the UK Ministry of Housing, Communities & Local Government^21^, to understand the socio-economic profile of customers and analyse correlations between deprivation levels and spending behaviour.

The Living Cost and Food Survey (LCFS), collected annually by the Office for National Statistics (ONS), provides detailed data on household expenditure, income, and demographics. The UK Multi-Region Input-Output (UKMRIO) dataset offers a model of the flow of goods and services across UK regions, detailing inter-industry interactions, consumption, and environmental impacts. Together, these datasets allow us to estimate the carbon emissions linked to different categories of expenditure^6^.

### Data integration and fusion

To integrate the financial transaction data with these government datasets, we follow a structured process. First, carbon emissions for each transaction are estimated using the third approach described in Trendl et al.^6^, which derives emissions from financial transaction data combined with MRIO-based carbon multipliers. Specifically, we apply carbon intensity multipliers developed by Trendl et al.^6^, based on the LCFS and UKMRIO datasets. These multipliers are linked to COICOP categories, which are then mapped to MCCs in the transaction data using established mappings^2,26–28^. This approach allows us to estimate emissions for each transaction based on its MCC and spending amount, with adjustments for inflation using Consumer Price Index (CPI) values.

Second, the IMD is linked to the transaction data using customer postcodes, matching each postcode to a Lower Layer Super Output Area (LSOA) in the IMD dataset. This provides deprivation statistics for each customer, enabling an analysis of socio-economic influences on spending behaviour and carbon emissions. We managed to map every customer to the respective level of deprivation of their environment.

By combining these datasets, we ensure a comprehensive analysis of customer spending patterns, socio-economic contexts, and associated carbon emissions.

### Bipartite network creation

Bipartite networks are used to represent systems consisting of two distinct types of nodes. In simple bipartite networks, connections only form between nodes of different types, which makes them ideal for modelling relationships in complex datasets. For example, in ecology, bipartite networks can illustrate interactions between species and their environments, helping researchers understand ecosystem dynamics^29,30^. Similarly, in recommendation systems, these networks can connect users with items, allowing for tailored recommendations based on user preferences^31^. In economics, bipartite networks can represent relationships between different economic agents, such as consumers and products, facilitating insights into market behaviour^32^.

By highlighting these applications, we can see that bipartite networks are not just theoretical constructs; they are a practical tool that helps us analyse and understand complex interactions between distinct groups across various fields. They can be used for data mining, pattern recognition, and identifying relationships within complex systems, making them useful in both research and applied contexts. In the context of this research, their role extends to identifying consumer spending patterns and estimating carbon emissions, thereby providing valuable insights for policymakers and financial institutions seeking to implement effective carbon reduction strategies. In this research context, these networks allow customers to be linked with the transaction categories they engage with.

**Fig. 2:**
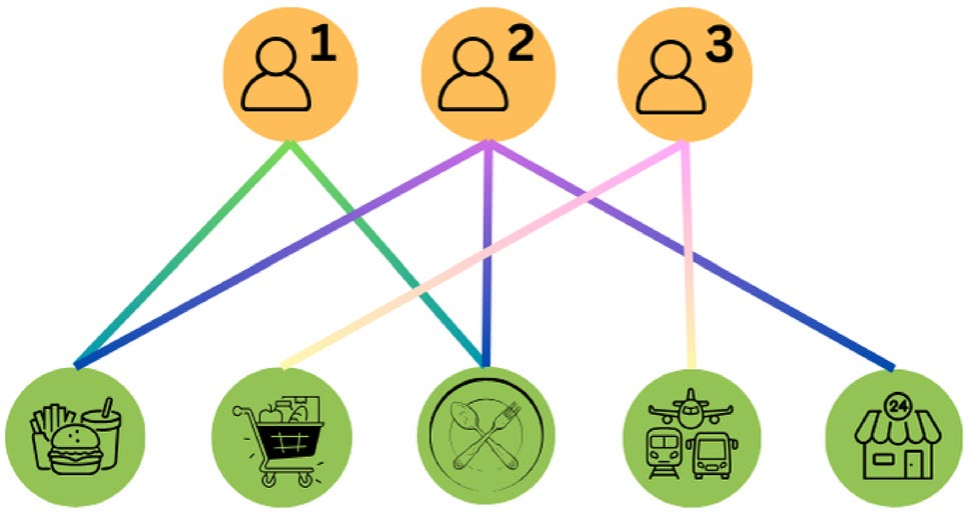
An example of a transaction bipartite network, showing the connection between customers (top) and MCCs (bottom). Each edge represents that the customer has had at least one transaction in that merchant category.

To understand customer spending behaviour, we construct a bipartite network with customers as one type of node and MCCs as the other. An edge is created between a customer and an MCC if the customer has made a transaction in that category. See Fig. 2 for an illustration of this bipartite network structure. This approach allows us to present all the data from a large transaction dataset in a single network, connecting customers and categories based on transaction patterns. We can then apply network analysis techniques to analyse the dataset, which provides additional insights compared to classical statistics and machine learning approaches.

This network approach also accommodates classification systems different from the MCC used by financial institutions, allowing for the exploration of connections and the identification of consumer communities based on their spending patterns. By incorporating various classifications, such as COICOP used by the UN and multiple policymaking institutions, this approach provides broad analysis of consumer behaviour across different contexts.

Additionally, we define two alternative edge-weighting schemes for the bipartite network to better capture different characteristics of the data. These weight assignments provide different ways to quantify the strength of the relationship between customers and merchant categories (MCCs) based on their transaction behaviour.

First, we use the number of transactions between a customer and an MCC as the edge weight. This means that for each customer-category connection, the weight is simply the count of transactions made by that specific customer in that category. This approach ensures that the network structure reflects not only the set of categories a customer transacts in but also the frequency of transactions within each category. By incorporating transaction counts directly as weights, we preserve the raw behavioural patterns without introducing external assumptions.

Second, we define an alternative weighting based on relative spending per category. Here, the edge weight represents the total amount a customer has spent in a given category, normalised by the average spending of all customers in that category. This normalisation ensures that spending behaviour is interpreted in the context of overall category trends, allowing us to identify customers who spend significantly more or less than average in a given category.

These weighting schemes do not introduce arbitrary modifications but rather directly derive from the transaction data itself–either as transaction counts or spending amounts–ensuring a transparent and interpretable network representation. By applying network analysis to these weighted structures, we uncover different aspects of consumer spending behaviour. The results of these analyses and their implications are explored in the *Discussion* section.

### Stochastic block modelling

A Stochastic Block Model (SBM) is a probabilistic model used to analyse the structure of networks by dividing nodes into distinct groups or “blocks” based on connection patterns. Its probabilistic nature makes it well-suited for community detection, allowing us to identify connection patterns within and between groups. Introduced in the 1980s by^33^, the canonical SBM views networks as structures composed of blocks of nodes. Connections between nodes are determined by their block memberships and predefined network parameters.

There have been many recent advancements in SBMs expanding their applicability to various networks across different scientific fields. Modifications to the canonical SBM structure include approaches that account for weights in networks^34^ and hierarchical communities^35^. Studies have also developed degree-corrected variations^36^ and overlapping communities^37^.

These models have been effectively applied to study community formation in various fields, such as in US Senate political cohesion and co-voting networks^38^, connections in healthy human gut microbiomes^39^, and relationships in ecology and ethnobiology^40^.

SBMs are particularly useful for analysing large-scale networks encountered in real-world applications, such as large financial transaction datasets. While our study focuses on UK consumption data, this approach is not limited to a single region. SBMs can be applied to transaction datasets from other countries, enabling cross-country comparisons of consumer behaviour and spending patterns by identifying similarities and differences in community structures across regions. Their high resolution limits allow for the identification of numerous communities with specific characteristics^35,41^. The probabilistic basis of SBMs ensures reliable community detection results based on observed data, making them an efficient tool for revealing insights into community organization in complex networks.

In contrast to methods like k-means or modularity maximisation, which may struggle with high-dimensional, sparse, or complex network data, SBMs are better suited for detecting meaningful community structures. SBMs identify statistically significant assortative modules by modelling the full probabilistic structure of the network, enabling them to avoid common pitfalls like resolution limits and overfitting^35,42^. Moreover, SBM’s hierarchical and nonparametric properties allow the detection of multi-scale community structures and fine-grained behavioural patterns without prior assumptions about the number or shape of the clusters^19^. This makes them particularly appropriate for analysing behavioural networks derived from financial transactions.

In this study, we apply a degree-corrected nonparametric hierarchical SBM on the bipartite network we previously described to identify communities of customers based on spending categories. We implement the model using Python and the graph-tool package. For reproducibility, the full code is openly available^24^. This specific SBM method was initially developed for topic modelling through word clusters in documents^43,44^. We apply this method to financial transaction datasets so that we can find communities of customers with similar spending behaviour in bipartite networks of customers and categories. Additionally, we introduce modifications to the code, so that we can use any arbitrary vector of weights between the customer and category nodes instead of solely relying on the number of repeated transactions as the weight.

However, due to its probabilistic nature, running the SBM algorithm only once does not guarantee finding the optimal partition consistently^19^. To address this variability, we run the algorithm 100 times, as this is typically sufficient for the entropy in the system to stabilise—meaning that the uncertainty in the community assignments reaches a consistent level. Stabilisation of entropy is desirable because it indicates that the algorithm has converged to a reliable partitioning of the network. However, we can run additional iterations if necessary. We then plot the change in entropy and select the iteration with the highest posterior probability. We choose the clusters generated by the SBM which are optimal because they achieve the lowest possible entropy, indicating a well-defined community structure.

##  Results

In this section, we present the findings from the SBM community detection applied to the bipartite network. We show how these communities vary based on the weights assigned within the network, providing insights into distinct patterns of consumer behaviour and their corresponding environmental impacts.

**Fig. 3:**
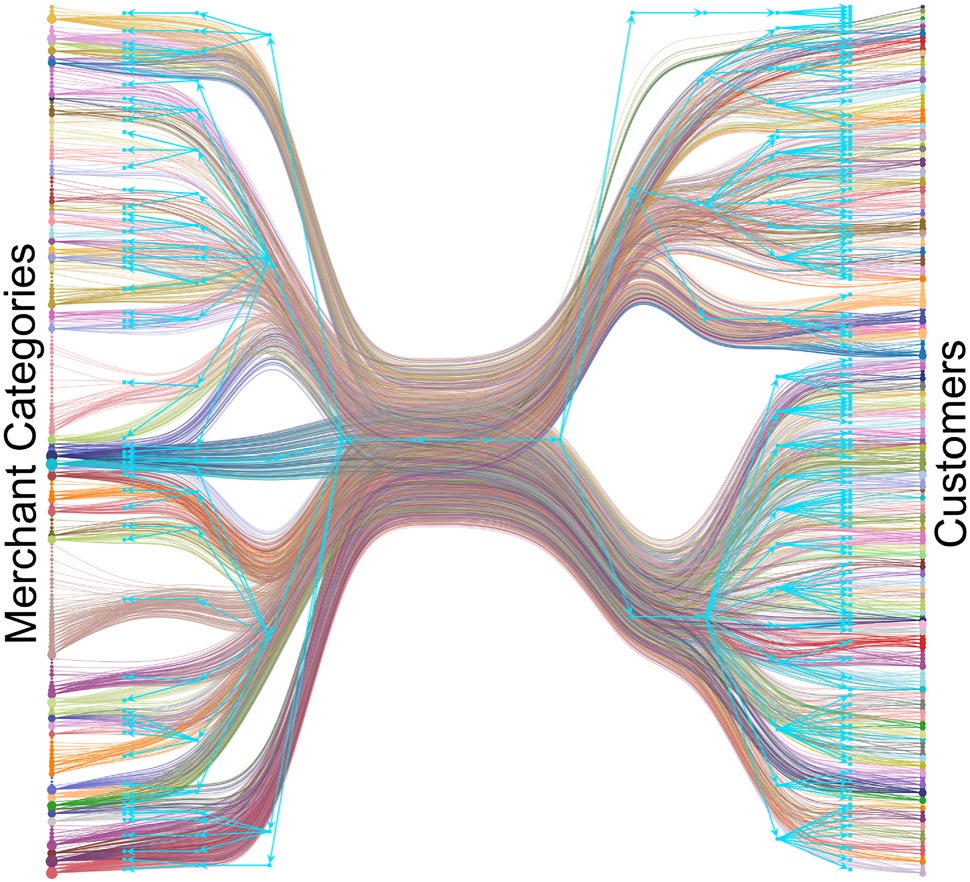
Community structure identified through SBM community detection on the bipartite network, with customer nodes on the right and merchant nodes on the left. Edges connect customers to specific transaction categories, and colours indicate different communities. The blue graph overlay shows hierarchical community formation, combining lower-level communities into larger groups, allowing analysis at varying levels of granularity.

### SBM community detection results

Applying the Stochastic Block Model (SBM) community detection to the bipartite network identifies clear groupings of customers based on their transaction patterns across specific merchant categories (see Fig. 3). These communities reflect clusters of customers who consistently engage with similar types of merchants, indicating shared spending behaviours and preferences.

We analysed the distribution of customer transactions within each community across all MCC categories. The results show that the transaction counts of customers within the same community are tightly clustered around the mean for each MCC, with minimal variation, as indicated by standard deviations close to zero. This statistical consistency in spending patterns suggests that customers in each community exhibit homogeneous behaviours. The uniformity across communities is supported by the low variance, providing clear evidence of distinct customer segmentation based on transactional behaviour.

The nested structure of the SBM we apply reveals hierarchical community structures based on consumer spending behaviours. This method allows for the identification of communities at various levels, beginning with the most granular communities at level 0, which are then aggregated into larger communities at higher levels. Within each higher-level community shown in the network, the nested SBM identifies sub-communities that share more specific spending habits. This relationship is illustrated in Fig. 2, where the different colours represent distinct communities and the blue graph overlay highlights the hierarchical nature of these communities, demonstrating how lower-level spending patterns contribute to the formation of broader groupings.

In this analysis, we focus on communities formed at level 1. This choice allows for a balanced understanding of customer behaviour, as level 1 communities in the case of this dataset capture significant spending patterns without becoming overly granular. Larger datasets may benefit from targeting lower-level clusters to limit the number of customers per community, while smaller datasets might be better suited for higher-level community analysis, focusing on broader groups of customers simultaneously. This flexibility helps ensure that we derive meaningful insights from the data at the appropriate level of detail.

**Fig. 4:**
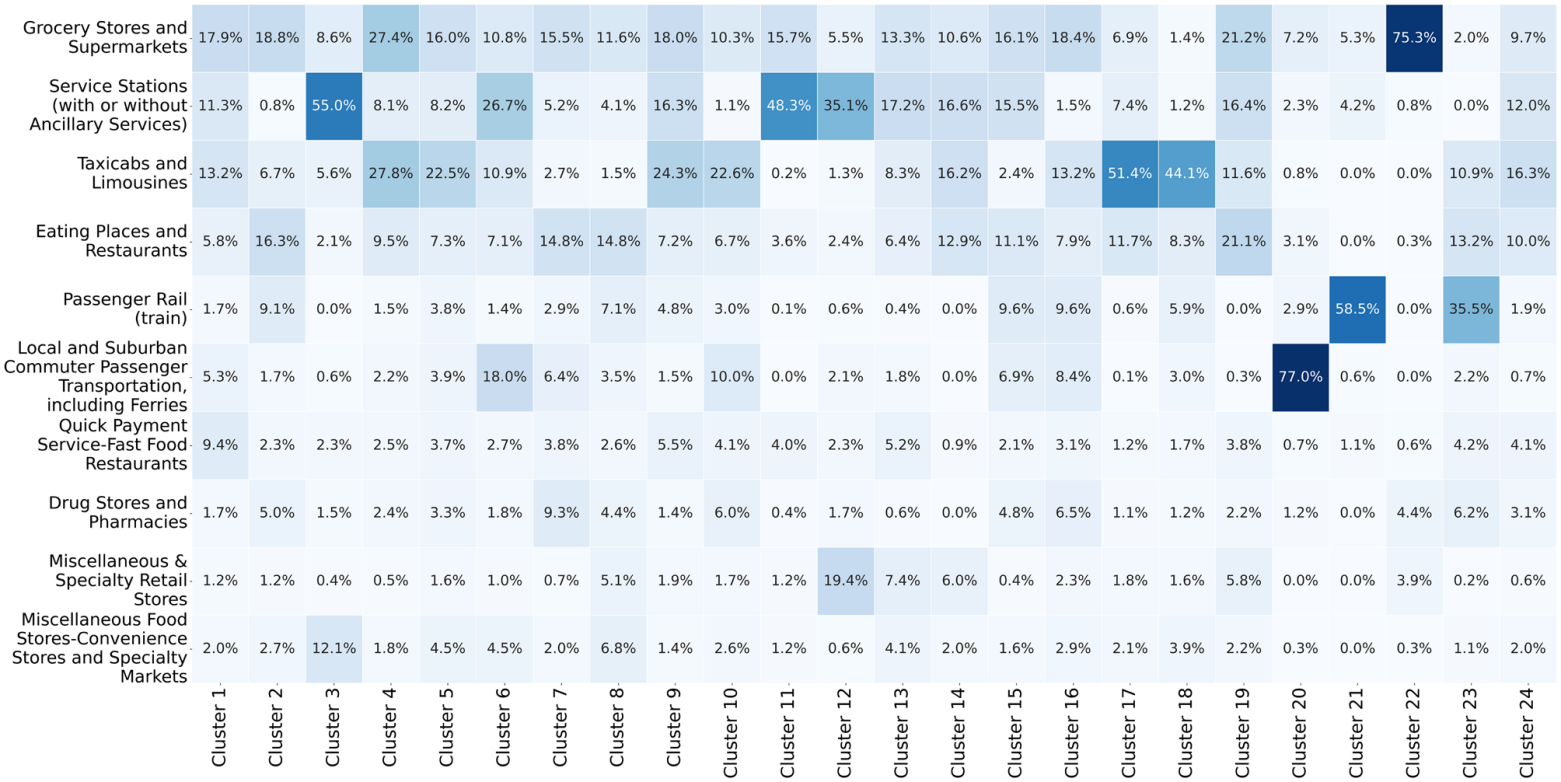
Heatmap with the percentage of carbon emissions per merchant category across customer clusters, showing the merchant categories that cause the highest and lowest emission levels for each cluster.

We analyse cluster attributes and find that certain merchant categories contribute disproportionately to the overall carbon emissions of each customer community. Specifically, we examine the percentage of total community emissions contributed by each category, as well as total spending and the number of transactions per category. For example, Fig. 4 shows the distribution of carbon emissions across merchant categories, highlighting the categories with the highest carbon footprint in each community. While certain clusters are driven by a dominant category in terms of emissions, others exhibit a more balanced distribution across several categories. Therefore, we report the emission shares of the top 10 highest-emitting categories in each cluster to offer a more complete picture of the emission profiles and enable comparison across different types of consumer behaviour. This analysis reveals the significant environmental impact of specific spending behaviours within the customer clusters.

The grocery stores category remains prominent in carbon emissions across most clusters because it is the most frequently visited category. However, Fig. 4 highlights that cluster 22 has a disproportionately high emissions tied to grocery spending, as customers in this cluster primarily use their cards for grocery purchases. Clusters 17 and 18 show elevated emissions linked to taxi usage, suggesting a heavier reliance on this mode of transport. Clusters 3, 11, and 12 exhibit significant emissions from service stations, indicating higher fuel consumption, while cluster 20 shows high emissions associated with public transport, reflecting the transportation preferences of consumers in that community.

These patterns may stem from two main factors. First, they likely reflect genuine spending behaviour, where customers naturally concentrate spending in categories relevant to their lifestyle, as with commuters in Cluster 20. Second, it may be influenced by how customers choose to use their *ekko* debit card, as incentives are based on transaction counts rather than amounts, leading some customers to use the card selectively for smaller or specific purchases while using other payment methods elsewhere. The observed clusters probably reflect a combination of these factors.

From a practical perspective, for customers near the minimum threshold of category diversity (i.e. 10 MCCs), *ekko* could introduce tools to encourage broader card use, such as rewards for diversifying spending or increasing transaction amounts in less-used categories. However, many clusters would capture authentic spending patterns, highlighting segments where targeted sustainability nudges and personalised communication focusing on dominant emission-driving categories can be particularly impactful.

By interpreting these clusters, we identify distinct consumer behaviours that contribute to varying levels of carbon emissions. For instance, certain clusters show a higher concentration of transactions in high-emission categories, such as gas station services and transport, which directly correlates with higher carbon footprints. Additionally, in Appendix 2, we provide the distribution of spending and the number of transactions across categories, further highlighting the spending patterns driving these emissions. This detailed breakdown reveals which consumer behaviours contribute most significantly to overall carbon output.

#### Weighted SBM results

In our analysis, we incorporate two types of edge weights to enhance the representation of customer behaviour in the bipartite network. The first type is based on the number of transactions between customers and MCCs, capturing the frequency of customer engagement with different categories. The second type reflects the relative amount spent by customers in each category compared to the average customer, helping us identify spending patterns and their potential environmental impact.

When incorporating the number of transactions as a weight, the resulting clusters exhibit varying carbon emissions across different categories. This highlights how transaction frequency influences emissions, providing insights into which categories contribute most significantly to a customer’s carbon footprint. For example, frequent transactions in high-emission categories may indicate opportunities for targeted interventions to promote sustainable alternatives.

When using spending amounts as weights, the SBM identifies clusters with consistent spending patterns across categories. These clusters reveal that customers within each group allocate on average similar proportions of their spending to different categories. As a result, the clusters themselves exhibit similar overall emission profiles, since the carbon intensity of each category is consistent across clusters. However, individual customers within a cluster may still have varying emission profiles due to differences in absolute spending levels or specific purchasing habits within a category.

To ensure spending and emission behaviours are comparable across categories, we normalise each transaction by dividing the amount spent by the average spending in that category. This allows for a more accurate comparison of customer behaviour and associated carbon emissions across categories with different average expenditures.

**Fig. 5:**
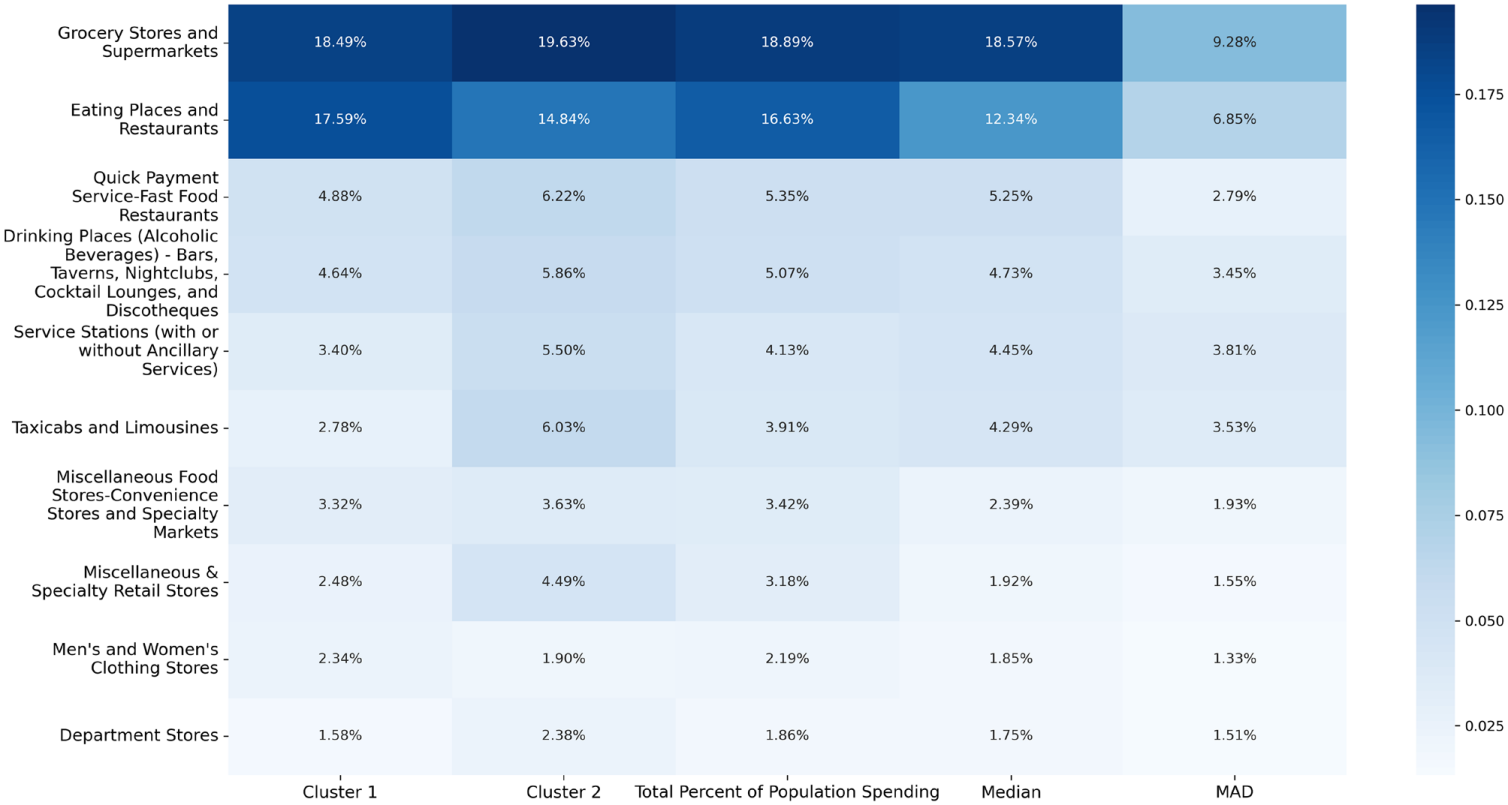
Heatmap illustrating the distribution of spending percentages of weighted SBM communities across different merchant categories. The cluster spending percentages (columns 1 and 2) fall within one median absolute deviation (columns 4 and 5) for each of the categories.

We visualise the spending patterns across different merchant categories using a heatmap (Fig. 5). The first two columns of the heatmap represent the customer percentage spending per community across various merchant categories, while the third column displays the average customer spending (population spending) for each category. The community spending is approximately equal across all 10 categories and closely aligns with the population values for each category. Since carbon emissions are derived using category-specific multipliers, equal spending across categories leads to similar emission profiles for each cluster, highlighting the relationship between spending patterns and environmental impact.

To show the consistency and central tendency across different customer groups, we examine if the cluster spending values are within one Median Absolute Deviation (MAD) of the median in the distributions of customer spending per category. The MAD is defined as the median of the absolute deviations from the median of the data set:

$$\text {MAD} = \text {median}(|X_i - \text {median}(X)|)$$

where $X_i$ represents individual data points. We use the median in this study as a measure of central tendency, as it is useful when dealing with outliers because it is not influenced by extreme values, unlike the mean. We typically observe a few outliers in most categories, as some customers concentrate their debit card usage on specific merchant categories, which skews the data.

When most values fall within one MAD of the median, it indicates that the data points are tightly clustered around the central value, demonstrating high consistency. However, as one MAD may seem an arbitrary choice, we also consider thresholds of 0.5 MAD to evaluate the similarity in cluster spending patterns further. In this context, a value close to the median implies that the clusters of customers exhibit similar spending habits, closely aligned with the population values.

**Fig. 6:**
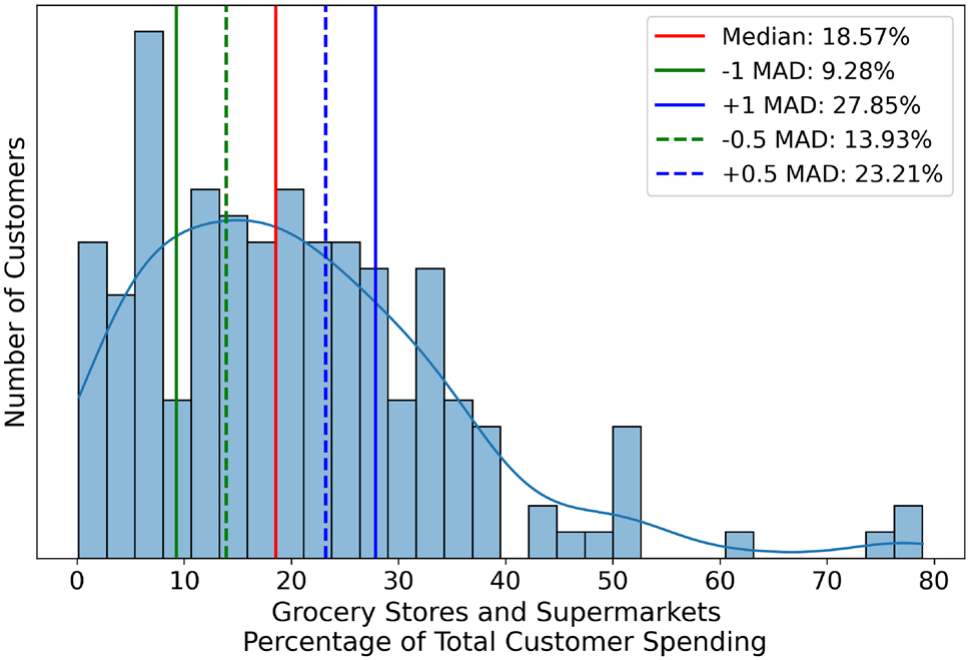
Percentage of total customer spending in the Grocery Stores and Supermarkets category with the median indicated in red and different MAD distances indicated with solid green and blue lines ($\pm 1$ MAD) and dashed green and blue lines ($\pm 0.5$ MAD).

We construct distributions of the percentage of total customer spending per category for all merchant categories and calculate the median and MAD for each distribution. One such distribution for Grocery Stores and Supermarkets is shown in Fig. 6. This represents a typical distribution observed across the categories. We include the median and MAD values for the ten most common spending categories in columns four and five of Fig. 5.

The spending percentages in the clusters across the ten most common categories are all within one MAD of the median, and 71 out of 80 clusters in categories with at least 30 customers fall within one MAD of the median. Moreover, we observe that approximately 3/4 of the clusters across the ten most common categories fall within half MAD of the median and 43 out of the 80 categories with at least 30 customers are also within half a MAD of the median. In comparison, when we apply this median-MAD analysis to the unweighted SBM communities, we find that fewer than half of the clusters fall within one MAD of the median and only about 1/5 of the clusters fall within half a MAD.

This indicates that the weighted SBM effectively identifies customer groups with consistent spending patterns, enabling targeted interventions to reduce emissions. For example, clusters with high spending proportions in high-emission categories could be prioritised for initiatives promoting sustainable alternatives, such as public transport incentives or renewable energy solutions. By focusing on both spending behaviour and emission intensity, this approach provides a robust framework for designing policies that encourage sustainable consumption at scale.

### Applying the model on larger datasets

Financial institutions and policymakers frequently deal with much larger datasets than the one used in our initial study. To evaluate the scalability and applicability of our approach, we generated an artificial larger dataset consisting of one million transactions and 3,000 customers that mirrors the distributions found in the original data. This synthetic dataset replicates key characteristics, including the number of transactions per customer, the distribution of Merchant Category Codes (MCCs) across transactions, the amount spent per MCC, and the corresponding emissions.

To simulate this dataset, we fit the parameters for probability density functions to the real data distributions for the number of transactions per customer, the distribution of MCCs across transactions, the amount spent per MCC, and we calculate the corresponding carbon emissions. We introduce joint distributions for correlated variables, such as spending amount and transaction frequency, to ensure that the relationships between these variables are accurately represented. After creating the specified number of artificial customer IDs, we draw values from the estimated probability density functions, ensuring that the generated dataset follows the distribution of the original data. We perform the Kolmogorov-Smirnov test to validate the accuracy of the synthetic dataset, which confirms that the generated distributions are closely aligned with the real ones.

When applying the SBM methodology to this larger dataset, we observe that the unweighted SBM continues to effectively identify clusters of customers with similar spending behaviours. However, in the context of larger transaction datasets, it may be more useful for financial institutions to focus on communities identified at the lower levels of the hierarchical SBM, which are more granular. These lower-level communities tend to contain a greater number of customers in cases of larger datasets, making them more relevant for large-scale analyses.

In the weighted case, applying SBM to the larger dataset identifies more communities. These communities maintain the pattern of equal percentage spending across MCCs observed in the original dataset.

In summary, our findings indicate that the methods used in this study are scalable and applicable to larger datasets, providing valuable insights for financial institutions and policymakers interested in analysing and mitigating the environmental impact of consumer spending. The analysis of smaller datasets (fewer than one million transactions) typically completes in seconds, while analyses of larger datasets with several million transactions, may take a few minutes or hours.

##  Discussion

In this section, we discuss the advantages and limitations of our approach, we compare our results to current literature, and highlight the ethical implications of developing spending behavioural incentives.

The results of our analysis can be used to identify customers with similar spending and carbon emissions patterns and to form communities with similar spending across merchant categories.

While the SBM does not take into account the socio-economic status of the customers when creating the communities, we show in Appendix 1 that these metrics are also important to consider. Therefore, we also briefly discuss the role of socio-economic metrics for behaviour interventions on a wider policy scale as well as on an individual business level.

### SBM community formation

The use of stochastic block modelling in our analysis helps mitigate bias by clustering customers based on network properties rather than demographic or socio-economic factors. The SBM identifies patterns in the data by examining the connections between customers and business categories, allowing for an unbiased grouping of customers with similar spending behaviours. This method ensures that the identified communities are based on actual transaction patterns, reducing the risk of reinforcing existing biases related to age, income, or other personal characteristics.

Using the results to target different groups of customers rather than individual customers is particularly advantageous when dealing with large datasets. By identifying distinct communities through SBM community detection, organisations can develop and implement group-specific strategies, optimising resource allocation and increasing the effectiveness of interventions. This approach is especially helpful in sustainable banking, as it allows banks to promote green financial products and services more effectively by targeting clusters of customers with similar spending habits, thus driving collective behaviour change towards sustainability. Additionally, it can be applied in marketing and customer relationship management, where tailored communication and incentives can be designed for each identified group, leading to improved customer satisfaction and loyalty.

The SBM algorithm offers significant advantages as compared to other community detection algorithms, particularly in the context of detecting community structures within large and complex networks^34,41,45^. Unlike traditional clustering algorithms such as k-means or mean shift clustering, which may struggle with the high-dimensional and sparse data, the SBM algorithm excels in these contexts by applying a probabilistic approach to identify clusters^38,42,46^. This allows the SBM to capture more detailed patterns and relationships between nodes, even in cases where other methods might fail to reveal meaningful groupings.

Communities identified through the SBM community detection offer valuable insights into how to target customers with similar carbon emissions patterns. By clustering customers based on their spending behaviours across different merchant categories, we can identify distinct groups that exhibit homogeneous emissions profiles, as indicated in Fig. 4. Then, for each community, we can analyse whether to develop incentives that target amount spent per transaction, number of transactions, or both.

**Fig. 7:**
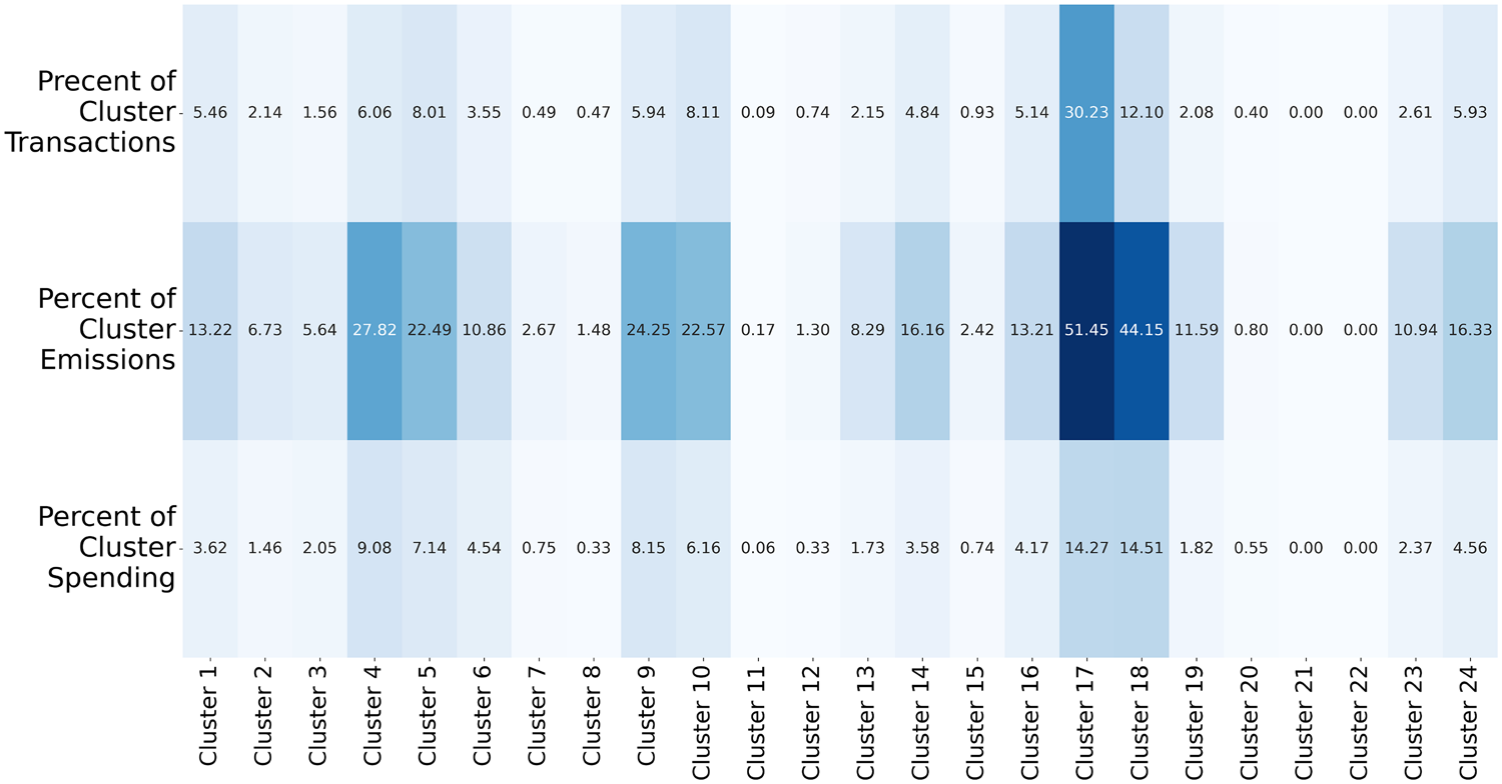
Identifying clusters with high percentage of emissions in the Taxicabs and Limousines category and analysing whether we should target amount spent per transaction or number of transactions.

For example, Fig. 7 shows the percent of cluster emissions for the Taxicabs and Limousines category and the respective cluster values for spending and amount of transactions in that category. Policymakers and financial institutions can identify groups of customers for whom spending in Taxicabs and Limousines causes highest share of emissions (clusters 17 and 18 in this case). Then, they can examine whether to target the number of transactions of that cluster (appropriate for cluster 17), amount of spending per transaction, or both (appropriate for cluster 18).

In behavioural science and economics, clustering customers based on consistent spending patterns while keeping other factors constant (ceteris paribus) is vital for understanding consumer behaviour. The weighted SBM allows for maintaining consistent spending across groups, enabling the analysis of the interaction of geographic, cultural, demographic, and socio-economic factors. This approach helps isolate these factors’ effects. For instance, in a multicountry study, researchers can compare consumer preferences based on geographic location or cultural background, eliminating the impact of varying spending behaviours. While the unweighted SBM identifies groups with similar spending patterns, applying total spending as an edge weight results in communities with spending patterns comparable to each other and the broader population. As demonstrated by the median and MAD analysis in Fig. 5, the weighted SBM effectively splits the customer dataset into communities with equal percentages spent per merchant category, aiding our understanding of consumer behaviour.

There are other studies that focus on analysing consumer behaviour through various methodologies, such as non-probabilistic clustering algorithms (k-means, hierarchical clustering, and partitioning around medoids)^47,48^, convolutional neural networks^49^, and association rule mining algorithms^50^. A convolutional neural network classifier uses visual representations of transaction histories, treating each transaction as a visual input that is processed through convolutional layers to extract features. This approach is primarily suited for tasks where spatial relationships within data (such as images) are crucial for classification or prediction. In contrast, our approach with the SBM community detection operates on the probabilistic dependencies inherent in transactional data.

Similarly, association rule mining focuses on discovering frequent itemsets and association rules, which may overlook the hierarchical relationships present in customer spending patterns that the SBM captures. Our research contributes to the existing literature by introducing a novel application of the stochastic block model to analyse consumer behaviour based on transactional data across multiple merchant categories. The SBM complements existing methodologies based on clustering algorithms because it operates on probabilistic principles derived directly from the data and has a much higher resolution limit for community detection.

Additionally, unlike other studies, we propose a method for preserving spending patterns across clusters of customers through the weighted SBM. The network construction can also be further adapted based on different needs. For example, constructing the network using COICOP categories instead of MCCs yields similar community structures based on broader economic expenditure classifications. This approach aligns with international economic classification and provides insights into consumer spending behaviours applicable for policy-making analysis.

However, the SBM approach has some notable limitations, including its static nature, computational expense, and increased difficulty in interpretation. The SBM is typically static and does not account for time-varying data or evolving consumer behaviour. For transactional data that changes over time, this limitation could affect the model’s ability to recognize changing patterns in spending behaviour. Additionally, the SBM relies on Bayesian probabilistic inference, which can be computationally expensive, especially with large datasets. This complexity is further compounded by the need to make specific choices regarding priors, for which there is not always a clear method, adding another layer of uncertainty to the modelling process. Moreover, the probabilistic framework can make the model harder to interpret and explain to non-technical audiences, limiting its practical usability in certain contexts.

### Importance of socio-economic factors

The community detection in the study is based only on the network we build from customer-category connections and their weights, so it does not directly take into account the consumers’ socio-economic status. This allows for an unbiased approach of identifying customers with similar spending patterns based only on their transaction history. However, when developing incentives for sustainable spending for different groups of customers, it is important to also consider the socio-economic status of the customers and how the incentive might affect their engagement with the banking institution.

In Appendix 1, we present the dependency of customer retention rates on the index of multiple deprivation and the age of the customers. Customers who are younger and live in more deprived regions tend to have higher drop out probability. The literature supports the finding that older customers are less likely to switch their banking provider^51,52^. The sustainable baking initiative we analyse also has a monthly fee to upkeep one’s account so this could present a barrier for customers from highly deprived areas to keep their accounts.

As different socio-economic metrics prove to be important for customer retention rates in paid banking initiatives, such institutions should take into account the socio-economic status of the consumer clusters they aim to incentivise, developing nudges that are tailored to their financial realities, such as flexible pricing and lower fees. These findings can also help policymakers to design inclusive financial policies that support diverse consumer needs and promote broader financial inclusion.

Wells et al.^18^ similarly highlight the role of demographics in shaping carbon footprints, showing that income, age, gender, and tenure influence the clustering of customers based on emissions. Their findings support our view that socio-economic variables should be incorporated into the design and targeting of behavioural interventions.

**Fig. 10:**
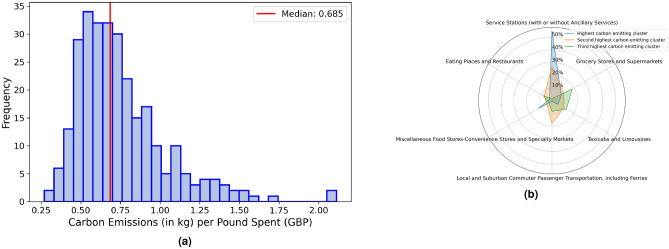
Panel (**a**) Distribution of customer carbon emissions per pound spent, showing a median emission rate of 685 grams per pound. (**b**) Spider diagram depicting the top three clusters with the highest emissions, identified using the unweighted SBM.

However, we find that once customers join the sustainable banking initiative, socio-economic measures have no significant effect on the carbon emission levels associated with them. This suggests that the initiative’s impact on carbon emissions is uniform across different socio-economic groups. The median value of carbon emissions in Fig. 10, derived from our sustainable banking dataset, is 685 grams per GBP. This figure is slightly lower than the values reported in other studies examining carbon emissions from large banking institutions^6^. This difference may result from multiple factors such as increased use of debit cards^53^ in the years between the studies, changing trends in consumer preferences after the COVID-19 pandemic^54^, and different spending habits between users of large banks and the smaller-scale baking initiative we focus on.

### Applications, policy implications, and ethical considerations

Behavioural nudging is designed to subtly guide customers towards specific behaviours without restricting their freedom of choice^55,56^. The results of our study can be used to nudge large groups of customers with similar spending habits, without having to develop specific incentives for each customer individually. By applying SBMs, we can detect communities of consumers who exhibit similar patterns of spending and associated emissions, allowing institutions to design community-level behavioural interventions. For instance, if a specific consumer cluster is characterised by unusually high spending on fuel or air travel, banking applications could display category-specific nudges to promote alternatives such as public transport, carpooling, or carbon offsetting. Unlike individualised approaches, this method allows for scalable interventions that maintain user privacy while still leveraging behavioural data to promote sustainability.

Additionally, the results of the weighted version of the SBM can help test the same nudges on consumers with similar purchasing profiles but different socio-economic status, geographic location, or demographic background. This allows financial institutions and policymakers to compare intervention effectiveness across groups that behave similarly in the marketplace but differ in external characteristics. Such comparison can inform the development of more fine-tuned and equitable behavioural incentives, allowing for context-sensitive adaptation of policies. For example, a nudge that successfully reduces emissions in a high-income urban cluster might be less effective or even counterproductive in a rural or lower-income group with limited mobility options. The segmentation produced by the weighted SBM creates a structure for not only targeting but also evaluating and adapting behavioural policies in a responsible and data-driven manner.

However, the application of behavioural nudges raises several ethical and societal considerations^56–58^. Nudges are meant to influence decision-making processes, often without explicit awareness. Even if the aim is to promote environmentally friendly behaviour, it is crucial to maintain transparency so customers are aware of how their decisions are being influenced, thereby preserving their autonomy. Equity and fairness are also essential in designing nudging strategies. There is a risk that nudges could disproportionately impact certain groups, especially if they are based on socio-economic indicators like income or age. For example, encouraging reduced spending on certain goods might be more challenging for lower-income customers, potentially exacerbating existing inequalities. Inclusive design and careful consideration are therefore necessary to ensure that nudging strategies do not disadvantage specific groups. Developing differentiated nudges for different socio-economic communities–while treating them with equal respect and concern–is an essential component of responsible behavioural policy.

Furthermore, while nudges can lead to short-term behavioural adjustments, their impact on long-term habits is less certain. Financial institutions and policymakers must evaluate whether their strategies foster genuine, lasting changes in behaviour or merely temporary compliance. Sustainable change is more likely to be achieved through a combination of nudging, educational outreach, and participatory engagement with communities. By addressing these broader societal implications, nudging can more effectively foster low-carbon consumer behaviour while maintaining trust and respect for individual freedoms.

In terms of broader implications, the findings from this research suggest that sustainable banking initiatives should adopt flexible, data-informed strategies for customer consumption targeting. The use of SBM allows institutions to identify coherent consumer groups whose purchasing patterns carry distinct carbon signatures. Rather than assuming uniform behaviour across an entire customer base, these communities enable targeted outreach and policy experimentation. Such segmentation supports not only efficient deployment of behavioural nudges, but also more inclusive and adaptive sustainability strategies. Policymakers, in turn, can use these insights to design fairer and more effective climate policies that take into account how consumption, and its environmental impact, varies across the population.

### Limitations of financial transaction data

Financial transaction data offer a valuable lens into consumer behaviour, but their use is subject to several important limitations that affect policy relevance. Access to such data is often constrained by privacy concerns and commercial restrictions^6^, which limits the availability of representative datasets and poses barriers to broader policy applications. Another significant limitation to the use of transaction datasets is that current research depends on samples derived from a single bank or financial institution, which may introduce biases, omitting customers who primarily bank elsewhere or remain unbanked^18^. This also applies to our dataset, as it reflects a sustainability-oriented subsample of the population, limiting representativeness compared to the general population. In our data, also key categories like utility payments and cash expenditures are often missing or underrepresented, as many such payments occur via direct debit or cash, which are not captured comprehensively in transaction data.

Additionally, carbon footprint estimates based on spending data inherently assume uniform carbon intensity within merchant categories, which can mask important variations in product-level emissions^6^. Combining data from multiple financial institutions and other sources such as surveys can provide a more robust basis for analysis. Nonetheless, despite these limitations, transaction data remain a promising tool for scalable and dynamic carbon accounting, offering insights into spending behaviour on individual level.

## Conclusion

This study demonstrates the potential of the SBM for analysing consumer behaviour and carbon emissions in financial transaction data. By identifying community structures within customer data, SBM helps the creation of archetypal profiles that capture distinct spending habits and their corresponding environmental impact. These profiles provide valuable insights for banks, policymakers, and other institutions for creating targeted interventions that promote more sustainable consumption patterns and reduce carbon emissions.

The findings of this research provide a foundation for understanding how consumer behaviour relates to carbon emissions across various spending categories. By clustering customers with similar spending profiles, the study allows for targeted interventions and personalised strategies. Additionally, these clusters are associated with similar carbon emission profiles, highlighting groups of customers whose behaviour contributes to comparable environmental impacts. This approach provides institutions with a data-driven framework for designing interventions that encourage more sustainable consumption and carbon reduction.

While the current approach is based on a static bipartite network, future research could benefit from extending this model to dynamic and multi-level networks. Such models would be able to capture the evolving nature of consumer behaviour and the temporal changes in carbon emissions, providing more accurate insights over time. Incorporating a temporal dimension would allow for a better understanding of how consumption behaviours change and how interventions influence carbon emissions in the long term. Dynamic models would also offer insights into how consumer decisions evolve, refining strategies to drive sustainable behaviour in the long run.

In conclusion, this research establishes the SBM approach as a powerful tool for understanding consumer dynamics and facilitating data-driven interventions to reduce carbon footprints. By clustering customers based on spending and emission patterns, this study offers a comprehensive framework for studying consumer behaviour and its environmental impact. Future research incorporating dynamic, temporal analyses will further enhance the adaptability of these models, supporting more effective resource allocation and policy interventions to achieve sustainability goals.

## Figures and Tables

**Fig. 8:**
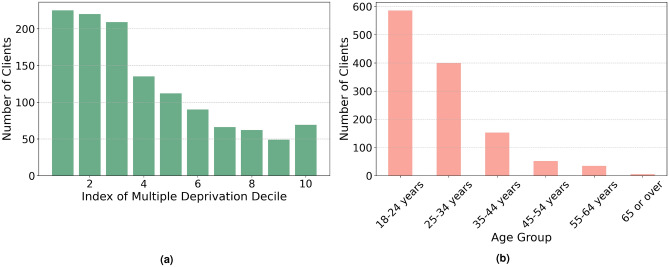
Distribution of client demographics in the financial transaction dataset. Panel (**a**) shows that, according to the Index of Multiple Deprivation, the majority of the customers are in the bottom three deciles, living in highly deprived areas. Panel (**b**) illustrates the distribution of clients across different age groups, showing that the customer base is predominantly younger. When compared to the national age distribution^63^, which tends to be more uniform, the age distribution of our customers highlights a significant concentration of younger individuals.

**Fig. 9:**
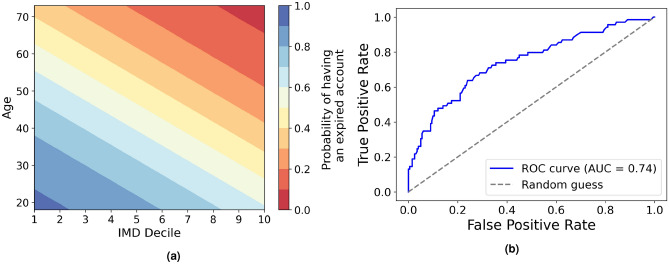
Panel (**a**): Logistic regression prediction for client retention/drop-out rates, showing that younger customer and customers who live in more deprived areas are more likely to drop out of the banking initiative. Panel (**b**): Receiver operating characteristic curve and AUC value corresponding to the model in (**a**), illustrating moderate discriminatory ability of the model.

**Fig. 11:**
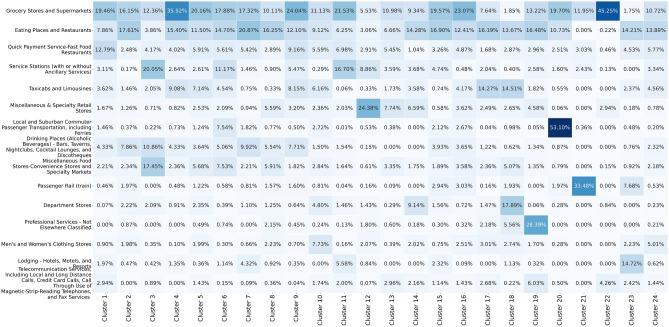
Heatmap showing the percentage of spending per merchant category across customer clusters, where darker shades indicate higher spending within each category and cluster.

**Fig. 12:**
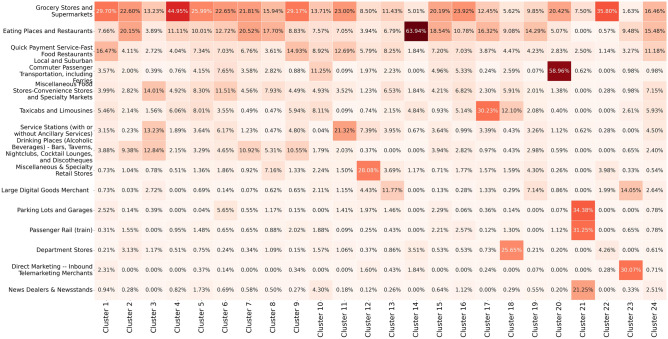
Heatmap showing the percentage of transactions per merchant category across customer clusters, where darker shades indicate higher numbner of transactions within each category and cluster.

## Data Availability

The publicly available data used in this study, including the Index of Multiple Deprivation (IMD), the Living Costs and Food Survey (LCFS), and the UK Multi-Region Input-Output (UKMRIO) data, can be accessed through the Office for National Statistics (ONS) database. The transaction dataset used in this research is confidential and cannot be shared due to data protection agreements.

## Appendix 1: Impact of socio-economic metrics on client retention and carbon emissions

In this appendix, we outline the socio-economic background and age of the customers in our financial dataset compared to the general UK population. We then develop a logistic regression model to predict client retention rates based on socio-economic and age metrics, highlighting the need for tailored emission-reduction interventions based on these factors.

The users of ekko^20^, the sustainable banking initiative, are predominantly younger people and individuals living in highly deprived areas (see Fig. 8). Research suggests that younger people are generally more environmentally conscious, which explains their strong interest in sustainable financial products^59,60^. Additionally, individuals from deprived areas may be more motivated to adopt eco-friendly practices due to higher pollution rates in their communities^61,62^.

We explore the impact of socio-economic metrics on client retention rates within the sustainable banking dataset. Understanding these factors can help financial institutions in designing effective incentives aimed at encouraging customers to modify their spending behaviors and reduce their emissions patterns. We also present total carbon emissions of customers and the differences between emissions per cluster.

We use a logistic regression model to predict client drop-out probability based on age and deprivation (as defined by the IMD Rank) values. The model achieves an accuracy of 0.78, indicating its ability to correctly classify clients into retained and dropped-out categories. According to the classification report, the model shows strong precision (0.78) and recall (0.97) for retained clients, suggesting good performance in identifying those likely to stay with the banking initiative. However, for clients likely to drop out, precision and recall are lower at 0.71 and 0.22, respectively, reflecting challenges in accurately predicting drop-outs. We obtain a weighted average F1-score of 0.73, suggesting balanced performance across both classes with some room for improvement in predicting drop-outs. The model has an Area Under the Curve (AUC) of 0.744 which indicates moderate discriminatory ability in distinguishing between retained and dropped-out clients based on the given features. These results underscore the significance of age and deprivation metrics in understanding and potentially mitigating client retention within sustainable banking initiatives.

We present the predicted client retention/drop-out probability in Fig. 9a and the respective receiver operating characteristic curve and AUC value in Fig. 9b. Individuals living in highly deprived areas and older individuals have a higher probability of dropping out of the sustainable banking FinTech.

We also examine the distribution of carbon emissions across customers (see Fig. 10a) in order to compare these values with other studies. We obtain a median value 685 grams of carbon emitted for every pound spent. We look at carbon emissions per cluster—panel (b) of Fig. 10 illustrates a diagram for the three clusters with the highest carbon emissions, identified using the unweighted SBM, showing the specific categories contributing most to these clusters’ carbon footprints. The visualizations in Fig. 10 highlight the variation in carbon emissions among customers and emphasize how emissions differ for clusters based on merchant categories.

## Appendix 2: Spending and transactions percentage heatmaps

We present the heatmaps with the percentage of transactions Fig. 11 and percentage of amount spent Fig. 12 per merchant category across customer clusters.

The heatmaps provide insights into customer preferences across different merchant categories, which is crucial for designing targeted sustainability initiatives. By analyzing the distribution of transactions and spending, we can identify clusters that prioritize sustainable or lower-emission categories. This understanding allows us to create tailored marketing strategies or interventions that encourage customers to shift their spending towards these categories, ultimately reducing carbon emissions.

Comparing both transaction volume and spending amounts across clusters can help pinpoint which customer segments have the most significant influence on emissions in specific MCCs. A cluster with a high percentage of transactions and a significant amount spent in high-emission categories can be prioritized for targeted interventions aimed at reducing carbon footprints. By focusing our sustainability efforts on these high-impact clusters, we can maximize the effectiveness of our initiatives and drive meaningful reductions in overall emissions.
